# Positive Bias Temperature Instability in SiC-Based Power MOSFETs

**DOI:** 10.3390/mi15070872

**Published:** 2024-06-30

**Authors:** Vladislav Volosov, Santina Bevilacqua, Laura Anoldo, Giuseppe Tosto, Enzo Fontana, Alfio-lip Russo, Claudio Fiegna, Enrico Sangiorgi, Andrea Natale Tallarico

**Affiliations:** 1 Advanced Research Center on Electronic System, Department of Electrical, Electronic and Information Engineering, University of Bologna, 47522 Cesena, Italy; claudio.fiegna@unibo.it (C.F.); enrico.sangiorgi@unibo.it (E.S.); 2 STMicroelectronics, SRL, Stradale Primosole 50, 95121 Catania, Italy; santina.bevilacqua@st.com (S.B.); laura.anoldo@st.com (L.A.); giuseppe.tosto@st.com (G.T.); enzo.fontana@st.com (E.F.); alfio-lip.russo@st.com (A.-l.R.)

**Keywords:** silicon carbide MOSFETs, threshold voltage instability, V_TH_ characterization, trapping/de-trapping mechanisms, defects, reliability

## Abstract

This paper investigates the threshold voltage shift (ΔV_TH_) induced by positive bias temperature instability (PBTI) in silicon carbide (SiC) power MOSFETs. By analyzing ΔV_TH_ under various gate stress voltages (V_Gstress_) at 150 °C, distinct mechanisms are revealed: (i) trapping in the interface and/or border pre-existing defects and (ii) the creation of oxide defects and/or trapping in spatially deeper oxide states with an activation energy of ~80 meV. Notably, the adoption of different characterization methods highlights the distinct roles of these mechanisms. Moreover, the study demonstrates consistent behavior in permanent ΔV_TH_ degradation across V_Gstress_ levels using a power law model. Overall, these findings deepen the understanding of PBTI in SiC MOSFETs, providing insights for reliability optimization.

## 1. Introduction

The rapid growth of renewable energy [1] and electric vehicles (EVs) [2] is driving the development of power devices based on wide bandgap (WBG) semiconductors. Renewable energy sources such as solar and wind energy require efficient solutions to convert and manage electricity [3], as well as electric vehicles requiring high-power reliable semiconductor devices to control electric motors and charging systems [4].

Silicon carbide (SiC) stands out in the realm of power electronics, offering a robust and high-performance alternative to conventional silicon (Si) counterparts [5], thus representing one of the best choices for applications where high power and reliability are required, such as solar inverters, wind turbine control systems, and electric vehicle motor control systems.

SiC’s inherent properties enable devices to operate at higher voltages, maintain stability at elevated temperatures, and switch at high frequencies. In particular, the breakdown electric field strength, nearly ten-fold that of silicon, and a band gap three times wider [6], allow for operation at elevated voltages and temperatures. Another key advantage of SiC lies in its thermal performance; it can maintain consistent operation even under high-temperature conditions [7], which is crucial for many industrial and automotive applications. The high thermal conductivity of SiC also aids in mitigating temperature-dependent degradation, ensuring longevity and reliability.

The high-frequency operation capability of SiC devices enables more compact power electronics systems [8], offering higher power density and reduced cooling requirements and opening a spectrum of possibilities in various sectors, from power systems to switch-mode power supplies and EVs [9].

However, while SiC technology offers significant benefits, different challenges are still present, including intricate production processes, resulting in elevated costs, and notably, issues related to device reliability.

One reliability challenge is the lower short circuit tolerance of SiC devices compared with Si ones [10,11]. This necessitates the use of fast-acting gate drivers to ensure device safety and reliability. In addition, SiC devices have been observed to exhibit larger threshold voltage (V_TH_) instability compared with their Si counterparts, with a tendency to faster recovery [12,13,14]. In [15], two distinct trapping mechanisms contributing to V_TH_ shift (ΔV_TH_) have been identified under gate bias stress tests, i.e., trapping of charges in the near-interface oxide traps (also referred as border traps) and in intrinsic defects at the SiO_2_/SiC interface.

The presence of pre-existing border traps has also been investigated in [16,17,18,19,20], highlighting the role of the tunneling in the charging and discharging processes [19], and measuring capture and emission times in the order of μs [20]. The role of fast trapping mechanisms related to pre-existing interface defects has been analyzed in [21,22,23,24]. 

In addition to interface and border defects, the creation of new traps and/or the charge trapping in deeper energy-level defects, both localized within the oxide, has been demonstrated in [25] by applying a relatively large gate voltage.

Further investigations have indicated the role of the testing methods on the observed ΔV_TH_. In particular, the influence of positive/negative bias temperature instability (P/NBTI) on the electrical characteristics of SiC MOSFETs has been thoroughly studied using both slow and fast measurement techniques [26].

Recently, we reported a distinct temperature dependence of ΔV_TH_, which varies based on the measurement technique employed [27]. When using a slow-PBTI procedure, the effect of fast interface and border traps is not accounted for in ∆VTH, as their recovery time is shorter than the V_TH_ characterization time. As a result, the oxide charge trapping dominates ΔV_TH_, resulting in a positive temperature dependency, i.e., the higher the temperature, the greater the charge trapping, the higher ∆V_TH_. Conversely, a negative temperature dependency is observed when a fast-PBTI test is adopted, emphasizing the role of a fast interface and border traps in the overall behavior [27].

In this work, the ∆V_TH_ of SiC MOSFETs induced by different PBTI test procedures suggested by JEDEC JEP184 [28], here named transistor and diode modes, has been investigated. The role of the gate bias level on the different underneath trapping mechanisms has been analyzed. 

## 2. Devices under Test (DUTs) and BTI Characterization Techniques

**Figure 1:**
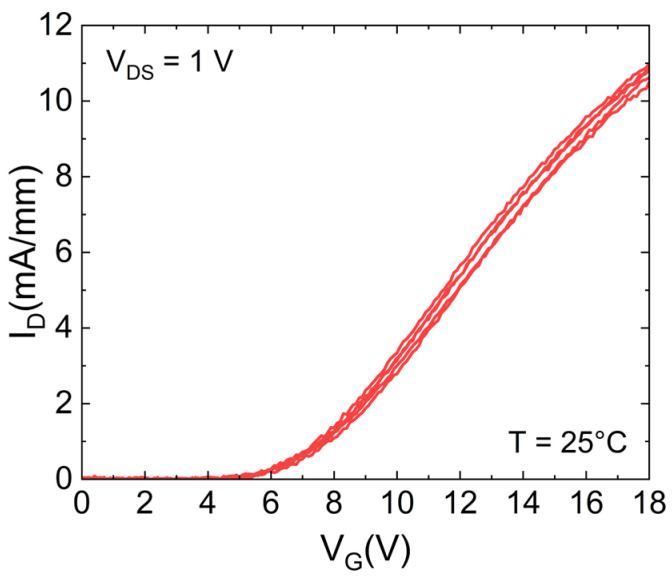
I_D_–V_G_ transfer characteristics of SiC MOSFETs with V_G_ sweep from 0 V to 18 V, V_DS_ = 1 V and ambient temperature T = 25 °C.

In this study, a 650 V automotive grade silicon carbide power MOSFET with a vertical-diffused structure (VD-MOSFET), manufactured by STMicroelectronics, has been considered. The room temperature transfer characteristics is reported in Figure 1, additional key features can be found in [29].

The Keysight Power Device Analyzer B1505A has been adopted for this analysis.

**Figure 2:**
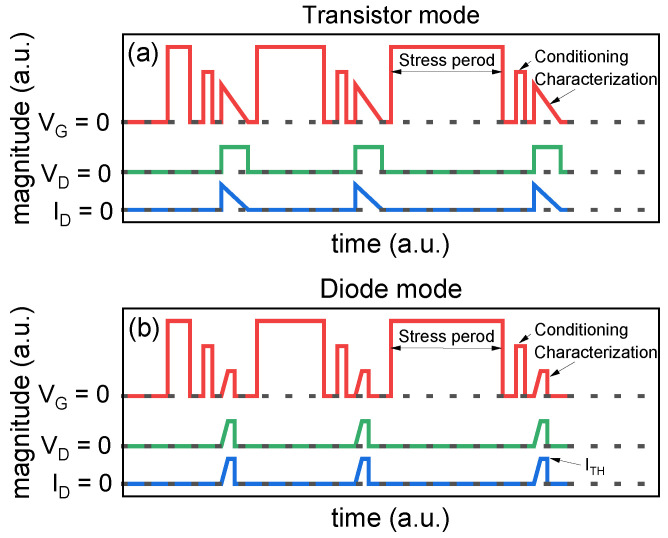
Waveforms of PBTI stress and measure procedure in the case of transistor (**a**) and diode mode (**b**) method, as reported by JEDEC JEP184 standard [28]. Each cycle consists of a logarithmically increasing stress period, conditioning and threshold voltage measurement.

Initially, a PBTI stress and characterization procedure according to the JEDEC standard JEP184, namely transistor mode, has been adopted and reported in Figure 2a. It illustrates the gate voltage (V_G_), drain voltage (V_D_), and drain current (I_D_) for the initial three stress and characterization periods. The gate maintains a steady bias during the stress phase, while the drain and source are grounded. Stress time periods, which increase logarithmically, are interspersed with V_TH_ sensing intervals. Following each stress interval, the gate stress is removed to allow for conditioning and V_TH_ sensing. To stabilize the V_TH_ readout, a conditioning phase is carried out by a 100 ms long positive gate pulse before the V_TH_ measurement. For the extrapolation of V_TH_, the I_D_–V_G_ transfer characteristics are measured. During this process, V_D_ remains constant whereas V_G_ is swept from V_G_MAX_ to 0 V to minimize V_TH_ recovery. V_TH_ is calculated at fixed I_D_ = 1 mA.

However, as ΔV_TH_ can be induced by slow and fast trapping/de-trapping components [16,17,18,19,20,21,22,23,24,25], slower measurements might result in the partal loss of the contribution ascribed to faster defects, i.e., fast defects recover before and/or during the V_TH_ characterization phase, thus not contributing to it. To gain a clearer understanding of these fast components, it is necessary to use faster measurement techniques.

Standard JEP184 also provides the gated-diode method for measuring the V_TH_ of SiC power transistors under BTI stress conditions. It involves biasing both the V_G_ and V_D_ simultaneously while maintaining the source at the ground potential. The test consists of two blocks: a stress phase for a specified period and V_TH_ characterization.

Similar to the previous method, during stress, V_G_ stress is applied to the gate terminal. The increasing gate stress time corresponds to a logarithmic scale.

The V_TH_ measurement method follows the JEDEC standard JEP183 [30], shown in Figure 2b. Firstly, as for the previous method, a gate conditioning pulse is applied, then V_TH_ of the SiC power MOSFET is measured in diode mode, which consists of the shorting gate and drain. The instrument forces the target threshold current (I_TH_), which determines the V_TH_ with a faster spot measurement (10 ms) compared with the full I_D_V_G_ characterization (few seconds), therefore avoiding V_TH_ recovery as much as possible.

## 3. Results and Discussion

**Figure 3:**
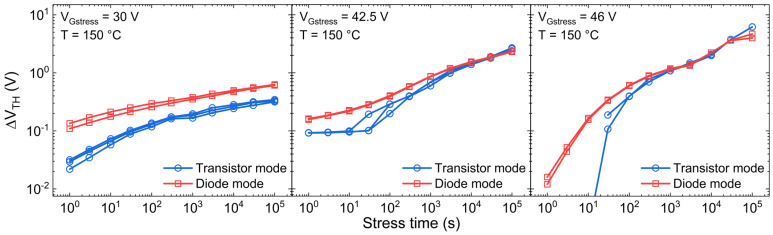
∆V_TH_ during the stress time by means of transistor (blue) and diode mode (red) technique, under different gate stress voltages and T = 150 °C.

Figure 3 reports the ΔV_TH_ under different gate stress voltages (V_Gstress_) at an ambient temperature of 150 °C. Notably, the ΔV_TH_ obtained by means of the diode mode approach is higher, especially for lower V_Gstress_ values (i.e., 30 V), although the stress phase is the same. The difference is ascribed to the different characterization phase, which is temporally shorter in the case of diode mode, allowing for a smaller V_TH_ recovery, hence capturing a larger ∆V_TH_. The difference between the two methods becomes more pronounced when operating at lower V_Gstress_ settings or for shorter stress durations. This is because the trapping and de-trapping processes in/from shallow pre-existing defects, which demand less time to capture and release charges, emerge as the predominant mechanism responsible for ΔV_TH_. As the gate voltage and stress time increase, the creation of new defects or the trapping in spatially deeper oxide defects starts to play a significant role, producing a permanent or slowly recoverable ∆V_TH_. As a result, the different characterization time that distinguishes the two methods no longer has an impact on the ∆V_TH_, as the recoverable part is negligible with respect to the permanent one.

**Figure 4:**
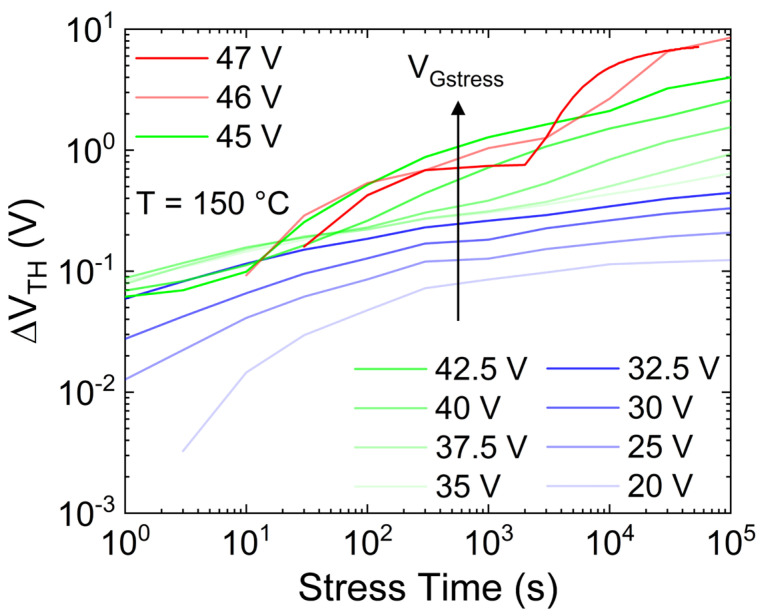
∆V_TH_ during the stress time as a function of different V_Gstress_, monitored by the transistor mode method, with T = 150 °C.

To demonstrate the occurrence of an additional mechanism (creation of new oxide defects or trapping in spatially deeper defects) with respect to trapping in the pre-existing defects, the PBTI analysis is performed at different V_Gstress_ ranging from 20 V to 47 V; the latter is a few volts below the breakdown voltage. Figure 4 shows ΔV_TH_ versus the stress time as a function of different applied V_Gstress_. It is possible to note the following: (i) for V_Gstress_ up to 32.5 V, the long-term ∆V_TH_ shows signs of saturation. This confirms the trapping in pre-existing defects with a finite concentration; (ii) from V_Gstress_ = 35 V to V_Gstress_ = 45 V, the ∆V_TH_ shows a second (higher) slope, indicating the triggering of an additional trapping mechanism, which occurs at shorter stress times by increasing V_Gstress_; (iii) for V_Gstress_ > 45 V, i.e., close to breakdown voltage, further trapping mechanisms seem to show up producing a further ∆V_TH_ slope variation. Moreover, under these high field conditions, a negative or smaller threshold voltage drift is observed for short stress times (<30 s), while a negligible V_Gstress_ dependency is observed for long stress times (>10^4^ s), indicating the presence of an additional competing mechanism, e.g., electron de-trapping from the oxide to the gate metal, contributing to V_TH_ decrease. Overall, by focusing on the long-term behavior reported in points (ii) and (iii), it may be ascribed to the creation of new oxide defects or charge trapping into spatially deep states, i.e., oxide traps far away from the SiO_2_/SiC interface.

**Figure 5:**
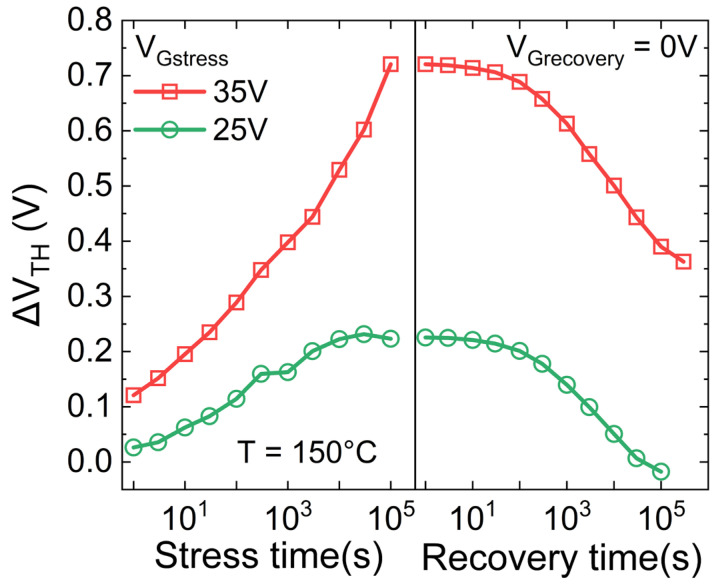
∆V_TH_ during the stress and recovery time as a function of different gate stress voltages, monitored by the transistor mode technique. Recovery condition: V_G_ = 0 V, T = 150 °C.

To strengthening this hypothesis, a stress test followed by the recovery phase has been carried out in the case of V_Gstress_ = 25 V and 35 V. Figure 5 reports a permanent or slowly recoverable ∆V_TH_, even after an extended recovery period of approximately 83 h at 150 °C, in the case of V_Gstress_ = 35 V, i.e., the bias condition in which ∆V_TH_ shows the occurring of a second slope. On the contrary, a lower stress level of V_Gstress_ = 25 V leads to a full recoverable ΔV_TH_ within just a few hours, confirming trapping and de-trapping in shallow pre-existing defects.

**Figure 6:**
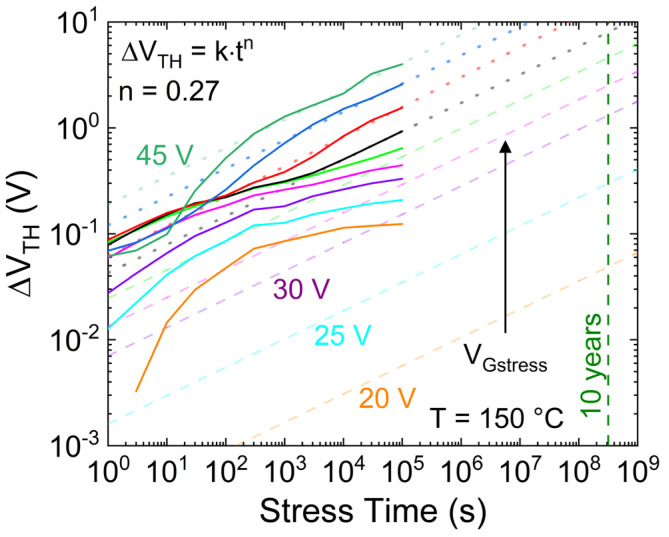
∆V_TH_ versus the stress time as a function of different V_Gstress_, monitored by the transistor method, with T = 150 °C. Solid lines: experiments. Dashed lines: ∆V_TH_ fitting by means of a power law, considering the second slope/mechanism observable for V_Gstress_ > 35 V. A 2.5 V voltage step has been adopted from V_Gstress_ = 30 V to V_Gstress_ = 45 V.

**Figure 7:**
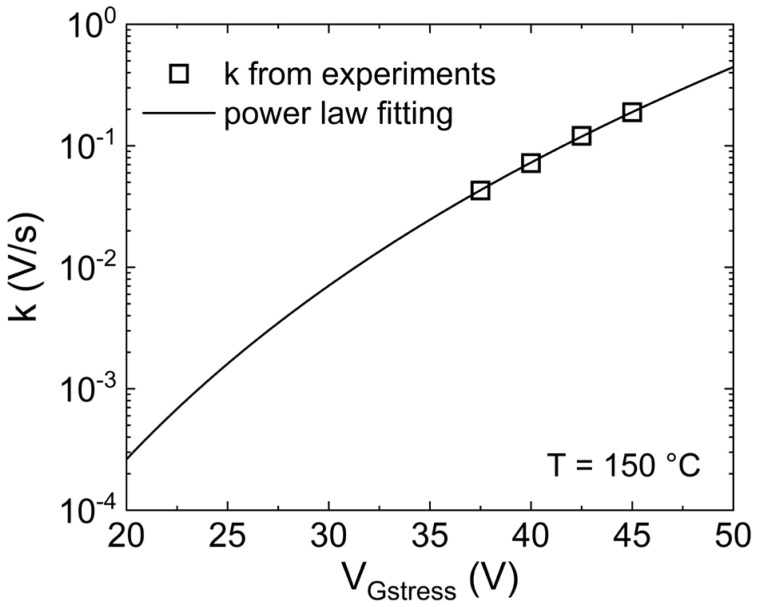
Scaling factor (k) dependence of the gate stress voltage. Symbols: k extrapolated from experiments (Figure 6). Line: fitting. The power law provides the smallest fitting error.

Focusing on the dynamics of ΔV_TH_ leading to permanent degradation (V_Gstress_ ≥ 35 V), it can model by using a power law, as shown in Figure 6. It is worth noting that ΔV_TH_ curves with V_Gstress_ > 45 V have not been considered because they are very close to the breakdown voltage. Therefore, the additional observed mechanisms are unlikely to occur under normal operating conditions. Figure 6 illustrates that the effect of the second mechanism on ΔV_TH_, whether it is creating new defects or trapping in spatially deeper states, always shows the same power slope (exponent) of n = 0.27, regardless of the gate stress voltage. Consequently, it is possible to assume that the same mechanism occurs even at lower V_Gstress_, but its impact is masked by trapping in the shallow pre-existing defects during the observed time windows. In particular, by obtaining the scaling factor k (symbols in Figure 7) through fitting the region of ΔV_TH_ experiments with steeper slope (dotted lines in Figure 6), the dependency of k on the gate voltage can be analyzed, resulting in a power-law relationship, as depicted in Figure 7. Consequently, the effect of this second mechanism can be estimated even at gate voltages closer to nominal operation (dashed lines in Figure 6) by deriving k from the model presented in Figure 7, utilizing n = 0.27. For instance, considering a maximum V_G_ = 25 V, the induced ∆V_TH_ due to the creation of new defects is estimated to be roughly 300 mV after 10 years at 150 °C.

**Figure 8:**
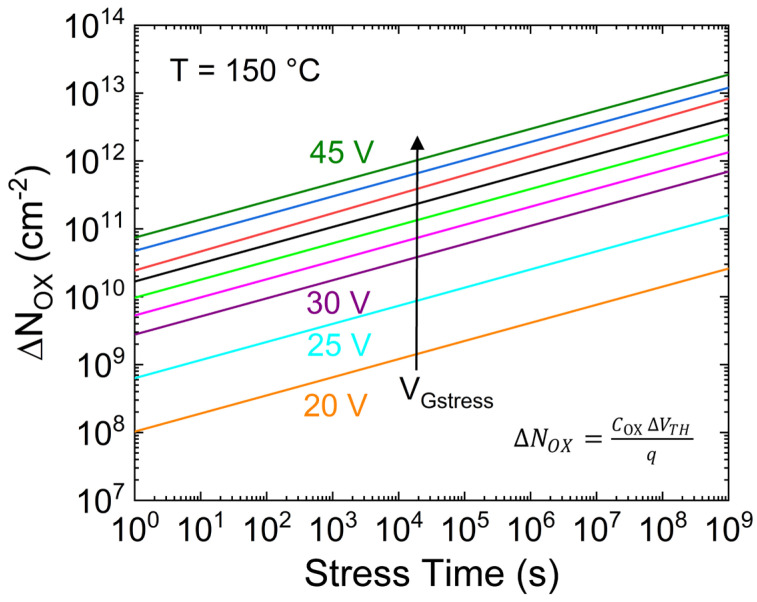
Oxide trapped charge density calculated from ∆V_TH_ data reported in Figure 6 (dashed/dotted lines).

By considering the ∆V_TH_ ascribed to the oxide charge trapping (i.e., dotted and dashed lines in Figure 6), the corresponding oxide trapped charge density (∆N_OX_) is calculated and reported in Figure 8 as a function of V_Gstress_. It is worth noting that the possible creation of new interface and/or border defects is excluded because, as demonstrated in [27], no degradation of the subthreshold slope has been observed (not shown).

**Figure 9:**
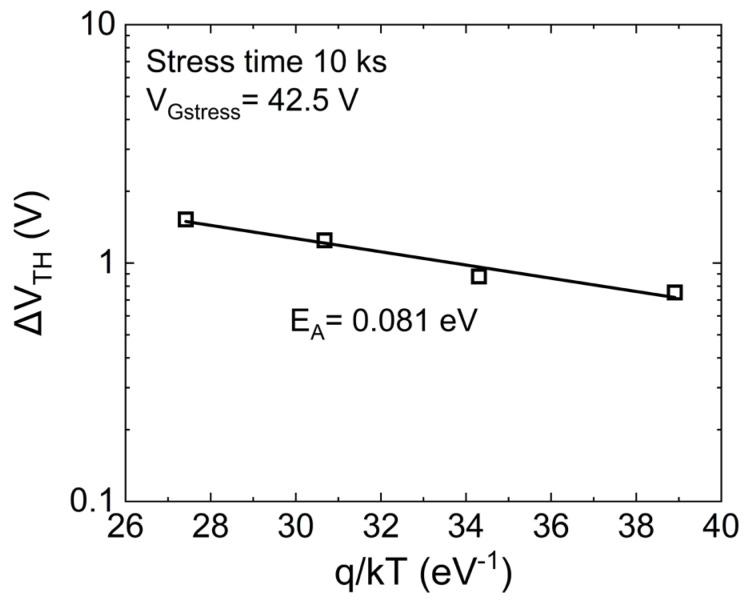
Arrhenius plot for the ∆V_TH_ measured after 10^4^ s of stress in the case of V_Gstress_ = 42.5 V.

Finally, a temperature-dependent PBTI analysis has been carried out to calculate the activation energy of the oxide traps inducing permanent or slowly recoverable ∆V_TH_, hence degradation_._ In particular, V_Gstress_ = 42.5 V has been adopted as it represents the bias condition in which the second ∆V_TH_ slope (trapping mechanism of interest) is clearly visible, whereas the short-term additional mechanism occurring at larger gate biases (close to the breakdown voltages) is almost negligible. As observed from the Arrhenius plot in Figure 9, such oxide defects feature an activation energy of ~80 meV. The relatively shallow energy level combined with the long recovery time (permanent) further confirms the creation of new oxide defects or the trapping in states far away from the SiC/SiO_2_ interface (spatially deep).

## 4. Conclusions

The positive bias temperature instability of SiC MOSFETs has been analyzed, revealing insights into the underlying mechanisms contributing to ΔV_TH_. The results demonstrate the importance of characterization methods, with the diode mode approach proving more sensitive to fast pre-existing defects compared with the transistor mode one, because of the reduced V_TH_ measure time, eventually leading to a smaller recovery. The analysis of ΔV_TH_ under different gate stress voltage conditions confirmed the presence of multiple trapping mechanisms, including trapping in pre-existing defects and the creation of new defects or trapping in spatially deeper states. These mechanisms exhibit distinct behaviors at varying V_Gstress_ levels, contributing to permanent or slowly recoverable ΔV_TH_. Overall, the findings contribute to a deeper understanding of the PBTI phenomena in SiC MOSFETs and provide valuable insights for enhancing device reliability.

## Data Availability

The original contributions presented in the study are included in the article.
